# Sex, age, role and geographic differences in traumatic spinal fractures caused by motor vehicle collisions: a multicentre retrospective study

**DOI:** 10.1038/s41598-023-30982-5

**Published:** 2023-03-06

**Authors:** Hong Yuan, Qin Guo, Zhixin Zhang, Lan Ou, Hongwei Wang, Hailong Yu, Liangbi Xiang

**Affiliations:** 1 Department of Orthopaedics, General Hospital of Northern Theater Command of Chinese PLA, Shenyang, 110016 Liaoning China; 2 Department of Outpatient, Xinqiao Hospital, Army Medical University, Chongqing, 400037 China; 3 Department of Orthopaedics, Sujiatun District Central Hospital, Shenyang, 110100 Liaoning China; 4 Department of Radiology, Southwest Hospital, Army Medical University, Chongqing, 400038 China

**Keywords:** Anatomy, Diseases, Health care

## Abstract

To investigate the sex, age, role and geographic differences in traumatic spinal fractures (TSFs) caused by motor vehicle collisions (MVCs) in adults (≥ 18 years old). This was a multicentre retrospective observational study. In total, 798 patients with TSFs caused by MVCs admitted to our hospitals from January 2013 to December 2019 were enrolled. The patterns were summarized with respect to different sexes (male and female), age group (18–60 and ≥ 60), role (driver, passenger and pedestrian) and geographic location (Chongqing and Shenyang). Significant differences in distribution related to district (*p* = 0.018), role (*p* < 0.01), motorcycle (*p* = 0.011), battery electric vehicle (*p* = 0.045), bicycle (*p* = 0.027), coma after injury (*p* = 0.002), pelvic fracture (*p* = 0.021), craniocerebral injury (*p* = 0.008) and fracture location (*p* < 0.01) were observed between the male and female groups. Significant differences in distribution related to district (*p* < 0.01), role (*p* < 0.01), car (*p* = 0.013), coma after injury (*p* = 0.003), lower limb fracture (*p* = 0.016), fracture location (*p* = 0.001) and spinal cord injury (*p* < 0.01) were observed between the young adult and elderly groups. Significant differences in distribution related to sex ratio (*p*  <  0.01), age (*p*  <  0.01), district (*p*  <  0.01), most vehicles involved (*P*  <  0.01), lower limb fracture (*p*  <  0.01), pelvic fracture (*p* < 0.01), fracture location (*p* < 0.01), complications (*p* < 0.01), and spinal cord injury (*p* < 0.01) were observed between the three different groups of pedestrian, passenger, and driver. Significant differences in distribution related to sex ratio (*p* = 0.018), age (*p* < 0.01), role (*p* < 0.01), most vehicles involved (*p* < 0.01), coma after injury (*p* = 0.030), LLF (*P* = 0.002), pelvic fracture (*p* < 0.01), craniocerebral injury (*p* = 0.011), intrathoracic injury (*p* < 0.01), intra-abdominal injury (*p* < 0.01), complications (*p* = 0.033) and spinal cord injury (*p* < 0.01) were observed between the Chongqing and Shenyang groups. This study demonstrates the age-, gender-, role- and geographic-specific clinical characteristics of TSFs resulting from MVCs and reveals a significant relationship between different ages, sexes, roles, geographic locations and associated injuries, complications and spinal cord injuries.

## Introduction

Traumatic spinal fractures (TSFs) are often associated with a significant impact on activities of daily living, resulting in a considerable socioeconomic burden^1–6^. Motor vehicle collisions (MVCs) are the main causes of TSFs, accounting for 15–67% in different studies^7–13^. TSFs have been discussed in many studies, especially TSFs caused by MVCs^14–21^. Age plays an important role in the pattern of TSFs resulting from MVCs^18–20^. Thoracic and lumbar spine fracture patterns are influenced by the age of the occupant. Extension injuries occur in older obese individuals and are associated with a high fatality rate^20^.

MVC is a serious health problem that results in associated injuries (ASOIs), such as orthopaedic fractures, craniocerebral injury, and thoracoabdominal injuries, and complications including deep venous thrombosis, pneumonia, pressure sores, urinary infection and postoperative infection. TSFs due to MVCs are commonly associated with orthopaedic fractures. This association occurs in distinct patterns and influences patient outcomes^15^. Thoracic and abdominal injuries account for 10.5% of MVC-related TSFs^16^. Traumatic spinal cord injuries (TSCIs) have a devastating effect on the quality of life of patients. Most TSCIs with TSFs caused by MVCs affect young patients and involve severe neurological impairments^21^. The characteristics of SCIs caused by MVCs have not been thoroughly studied, especially according to sex, age group, role and geographic location. Delays in the diagnosis of SCI and associated injuries (ASOIs), such as craniocerebral injury (CCI), intrathoracic injury (ITI) and intra-abdominal injury (IAI), may cause significantly increased morbidity and mortality.

In most of the studies on spinal fractures, researchers have simply reported that MVCs were the main cause of spinal fracture and indicated trends regarding related MVCs but have not discussed the specific epidemiological characteristics of the MVCs^7–13^. Reports from studies on spinal fracture caused by MVCs have included discussions of only some aspects of MVCs^14–21^, such as injured front-seat occupants^14^, paediatric passengers^18^ or elderly subjects^19^, traumatic spinal injury associated with orthopaedic fractures^15^, thoracoabdominal injuries^16^, and thoracolumbar fractures^17,20^. Although much is known about the characteristics of TSFs, TSFs caused by MVCs have not been discussed in depth according to different sexes, age groups, roles and geographic locations in a single study. In the current study, cases from multicentre tertiary hospitals in Chongqing and Shenyang that occurred between January 2013 and December 2019 were reviewed and analysed. The main purpose of the study was to investigate sex, age, role and geographic differences, especially in the pattern of ASOIs, spinal cord injuries and complications of TSFs caused by MVCs in adults (≥ 18 years old).

## Materials and methods

### Study population

We retrospectively identified 798 patients presenting to our hospitals with acute TSFs caused by MVCs between January 1, 2013, and December 31, 2019. There were 521 male and 277 female patients, with a mean age of 44.4 ± 13.7 years (range 18–90 years old) and a sex ratio of 1.88. Among the cases of TSF, 501 occurred in Chongqing, which is located in the southwest hot-moist region of China (the CQ group), and 297 occurred in Shenyang, which is located in the cool northern region of China (the SY group). Chongqing is the economic and financial centre of the upper Yangtze River and is mainly hilly and mountainous, with a large area of sloping land known as the “mountain city” and “foggy Chongqing.” It has a humid subtropical climate with annual temperatures ranging from 4 to 29 °C. The average temperature is 16–18 °C, and the annual precipitation is 1000–1350 mm. Shenyang is located in the cool northern region of China, the central part of Liaoning Province, extending mainly to the plains. It has a semihumid temperate continental climate, with annual temperatures ranging from − 35 to 36 °C. The average temperature is 8.3 °C, and the annual precipitation is 500 mm. The patients representing these cases were then categorized into a male group (n = 521) and a female group (n = 277) and a young adult group (n = 687) and an elderly group (n = 111) as well as driver (n = 333), passenger (n = 215) and pedestrian groups (n = 250). Data were collected from the General Hospital of Northern Theater Command, which is the largest military hospital in Northeast China (Shenyang), and Xinqiao Hospital and Southwest Hospital of Army Medical University, which are the two largest military hospitals in Southwest China (Chongqing). The hospitals where the patients were admitted are the main hospitals where patients with TSF and SCI are treated.

X-ray, computed tomography (CT) and magnetic resonance imaging (MRI) examinations were performed to make a definite diagnosis of spinal fractures. Data regarding sex (male and female), age (18–60 and ≥ 60), district (Chongqing and Shenyang), injury season (spring, summer, autumn and winter), role (driver, passenger and pedestrian), vehicle involved (car, motorcycle, truck, battery electric vehicle, bicycle, taxi, bus, tricycle, van), ASOI, complications, fracture location and spinal cord injury (SCI) were recorded. ASOIs included upper limb fractures (ULFs), lower limb fractures (LLFs), pelvic fractures (PFs), craniocerebral injury (CCI), ITI and IAI. Complications included deep venous thrombosis, pneumonia, pressure sores, urinary infection, and infection. Fracture locations included cervical, thoracic, lumbar, cervical + thoracic, cervical + lumbar, thoracic + lumbar and others. The American Spinal Injury Association (ASIA) scoring standard was used to assess SCI. Noncontiguous spinal fractures (NSFs) were defined as fractures in which there was at least one intact vertebra present between two injured or fractured vertebrae. The study was approved by the Ethics Committee of General Hospital of Northern Theater Command, and informed consent was obtained from all individual participants included in the study.

### Statistical analysis

All data were analysed using SPSS software (version 24.0, SPSS Inc., USA). The chi-square test or Fisher's exact test was used for the frequency data. Student’s t test was used to compare continuous variables between the two groups. One-way analysis of variance was used for comparisons among different groups. *P* < 0.05 was considered indicative of statistical significance.

### Ethical approval and informed consent

All procedures were in accordance with the ethical standards of the Institutional Research Committee and with the 1964 Declaration of Helsinki and its later amendments or comparable ethical standards. The study protocol was approved by the Ethics Committee of the General Hospital of the Northern Theater Command of the Chinese PLA.

## Results

### General characteristics of TSFs and TSCIs

The records of 798 patients who presented with TSFs resulting from MVCs between 2013 and 2019 were retrospectively reviewed (Table 1). There were 521 (65.3%) male and 277 (34.7%) female patients. The mean age of the patients was 44.4 ± 13.7 years (range: 18–90 years). Fractures most often occurred in the winter (26.6%) season. The roles were divided into driver (333, 41.7%), passenger (215, 26.9%) and pedestrian (250, 31.3%). The most common vehicles involved were cars (69.8%), motorcycles (11.5%), trucks (4.7%), battery electric vehicles (2.5%) and bicycles (3.3%). The most common fracture locations were the lumbar segment (287, 36.0%), cervical segment (244, 30.6%) and thoracic segment (175, 21.9%).

**Table 1:** Sex differences in TSFs caused by MVCs.

Data	Total	Male group	Female group	*P*
Total	798	521	277	
Mean age (years)	44.4 ± 13.7	43.2 ± 13.5	46.6 ± 13.9	
District				
Chongqing	501 (62.8%)	343 (65.8%)	158 (57.0%)	0.018
Shenyang	297 (37.2%)	178 (34.2%)	119 (43.0%)	
Age group				
18–60	687 (86.1%)	456 (87.5%)	231 (83.4%)	0.108
≥ 60	111 (13.9%)	65 (12.5%)	46 (16.6%)	
Injury season				
Spring	194 (24.3%)	132 (25.3%)	62 (22.4%)	0.825
Summer	193 (24.2%)	125 (24.0%)	68 (24.5%)	
Autumn	199 (24.9%)	127 (24.4%)	72 (26.0%)	
Winter	212 (26.6%)	137 (26.3%)	75 (27.1%)	
Role				
Drivers	333 (41.7%)	262 (50.3%)	71 (25.6%)	< 0.001
Passengers	215 (26.9%)	125 (24.0%)	90 (32.5%)	
Pedestrian	250 (31.3%)	134 (25.7%)	116 (41.9%)	
Vehicle involved	927	602	325	
Car	647 (69.8%)	418 (69.4%)	229 (70.5%)	0.803
Motorcycle	107 (11.5%)	82 (13.6%)	25 (7.7%)	0.010
Truck	44 (4.7%)	35 (5.8%)	9 (2.8%)	0.055
Battery electric vehicle	23 (2.5%)	10 (1.7%)	13 (4.0%)	0.029
Bicycle	31 (3.3%)	14 (2.3%)	17 (5.2%)	0.031
Associated injuries	389 (48.7%)	254 (48.8%)	135 (48.7%)	1.000
LLFs	65 (8.1%)	43 (8.3%)	22 (7.9%)	0.986
PFs	42 (5.3%)	20 (3.8%)	22 (7.9%)	0.021
ULFs	44 (5.5%)	24 (4.6%)	20 (7.2%)	0.168
Craniocerebral injury	102 (12.8%)	79 (15.2%)	23 (8.3%)	0.008
Intrathoracic injuries	214 (26.8%)	143 (27.4%)	71 (25.6%)	0.640
Intra-abdominal injuries	22 (2.8%)	14 (2.7%)	8 (2.9%)	1.000
Coma after injury	62 (7.8%)	52 (10.0%)	10 (3.6%)	0.002
Spinal cord injury	425 (53.5%)	234 (44.9%)	191 (69.0%)	< 0.001
NSFs	101 (12.7%)	67 (12.9%)	34 (12.3%)	0.901
Complications	35 (4.4%)	24 (4.6%)	11 (4.0%)	0.814
Deep venous thrombosis	7 (0.9%)	2 (0.4%)	5 (1.8%)	0.099
Pneumonia	22 (2.8%)	18 (3.5%)	4 (1.4%)	0.154
Pressure sores	4 (0.5%)	3 (0.6%)	1 (0.4%)	1.000
Urinary infection	2 (0.3%)	2 (0.4%)	0	0.773
Surgical site infection	3 (0.4%)	2 (0.4%)	1 (0.4%)	1.000

There were 389 patients (48.7%) who presented with ASOIs, 62 patients (7.8%) who presented with coma after injury, 35 patients (4.4%) who presented with complications, 425 patients (53.3%) who presented with SCI and 101 patients (12.7%) who presented with NSFs. The most common associated injuries were ITIs (214 cases, 26.8%), CCIs (102 cases, 12.8%) and LLFs (65 cases, 8.1%). The most common complications were pneumonia (22 cases, 2.8%) and deep venous thrombosis (7 cases, 0.9%). Using the ASIA classification, 107 patients (13.4%) exhibited ASIA A, 34 patients (4.3%) exhibited ASIA B, 47 patients (5.9%) exhibited ASIA C, 137 patients (17.2%) exhibited ASIA D, and 373 patients (46.7%) exhibited no neurological deficits (Table 1).

### Gender differences in TSFs

**Figure 1:**
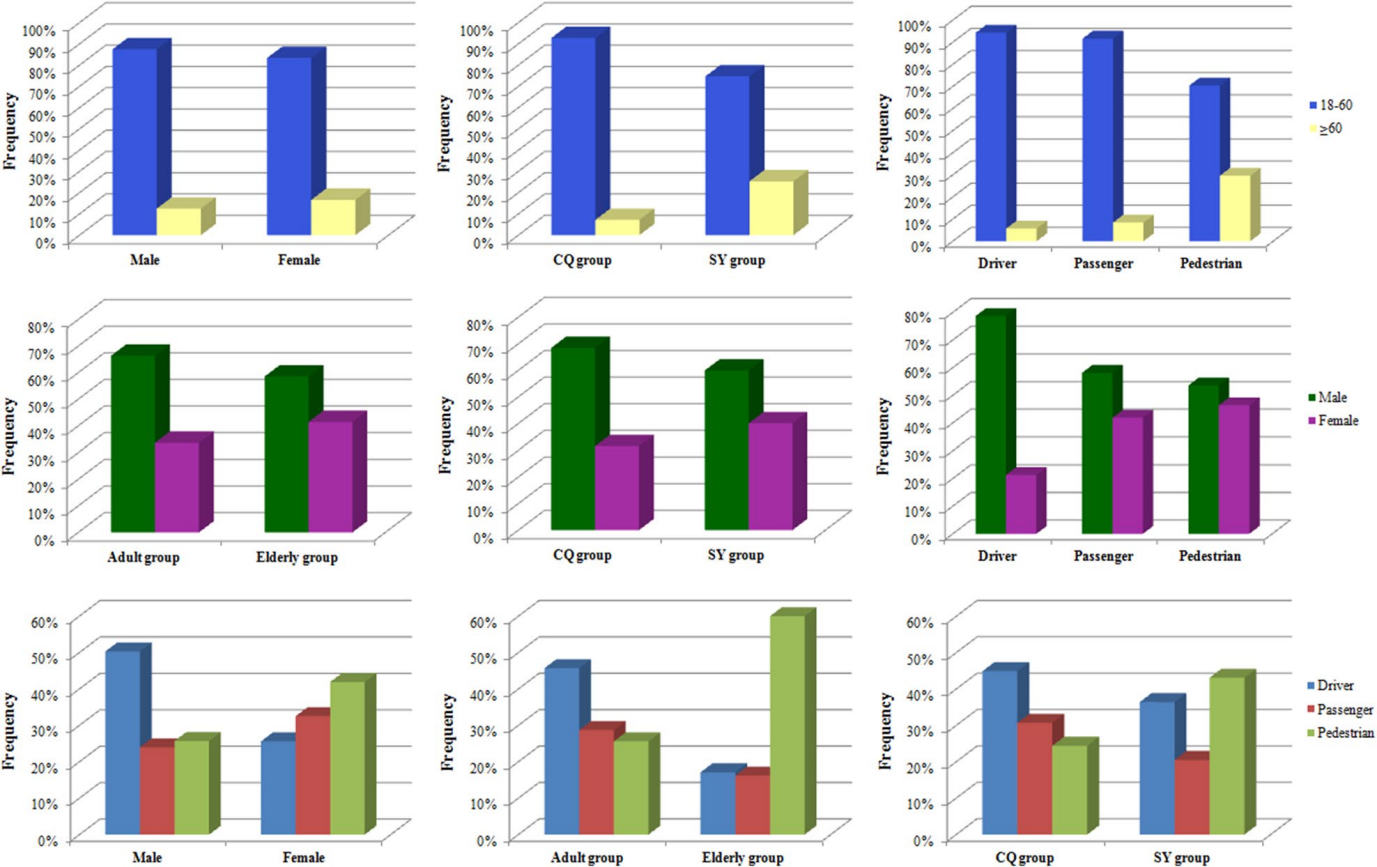
Distributions of age, sex and role according to different groups.

**Figure 2:**
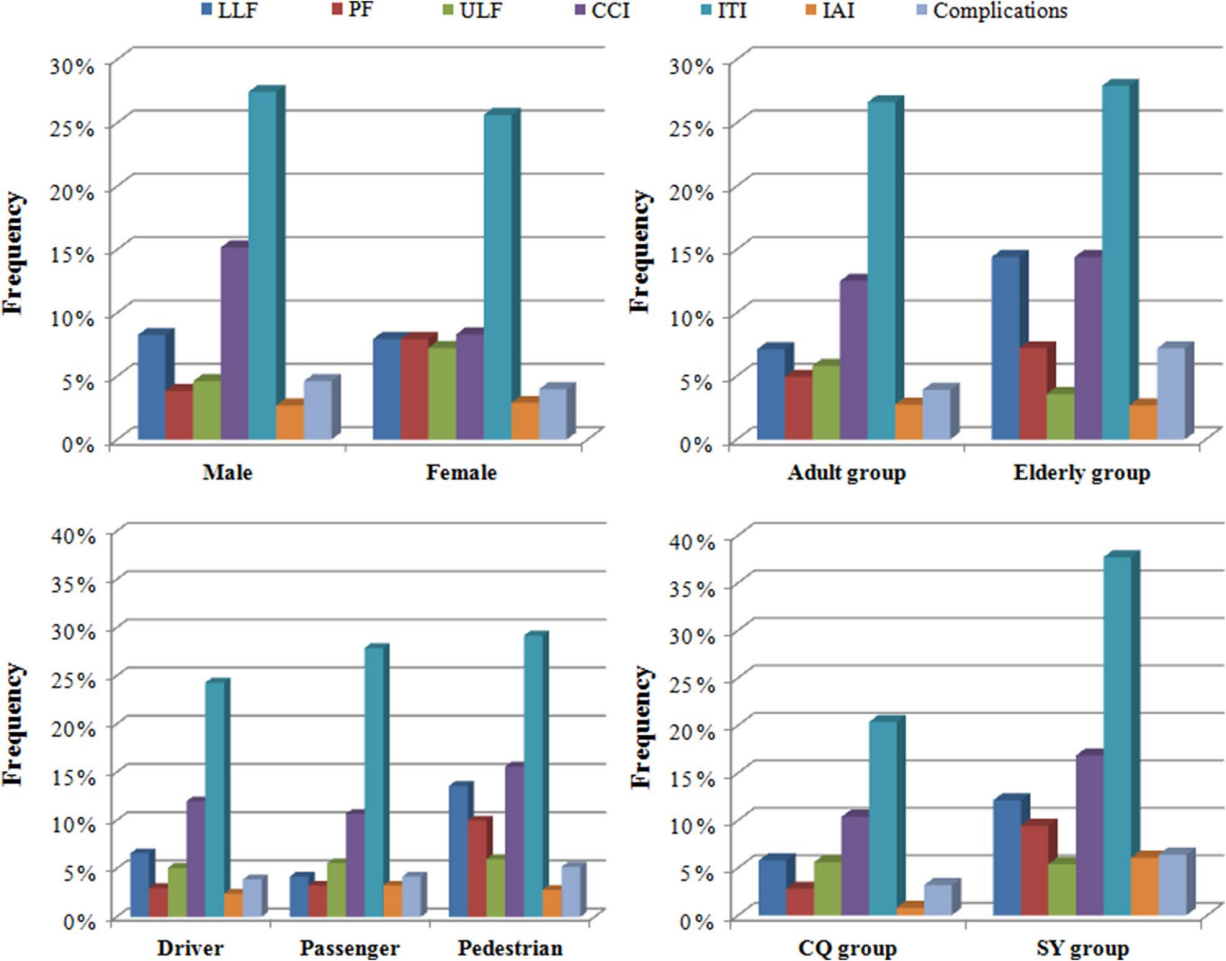
Distributions of associated injuries and complications according to different groups.

**Figure 3:**
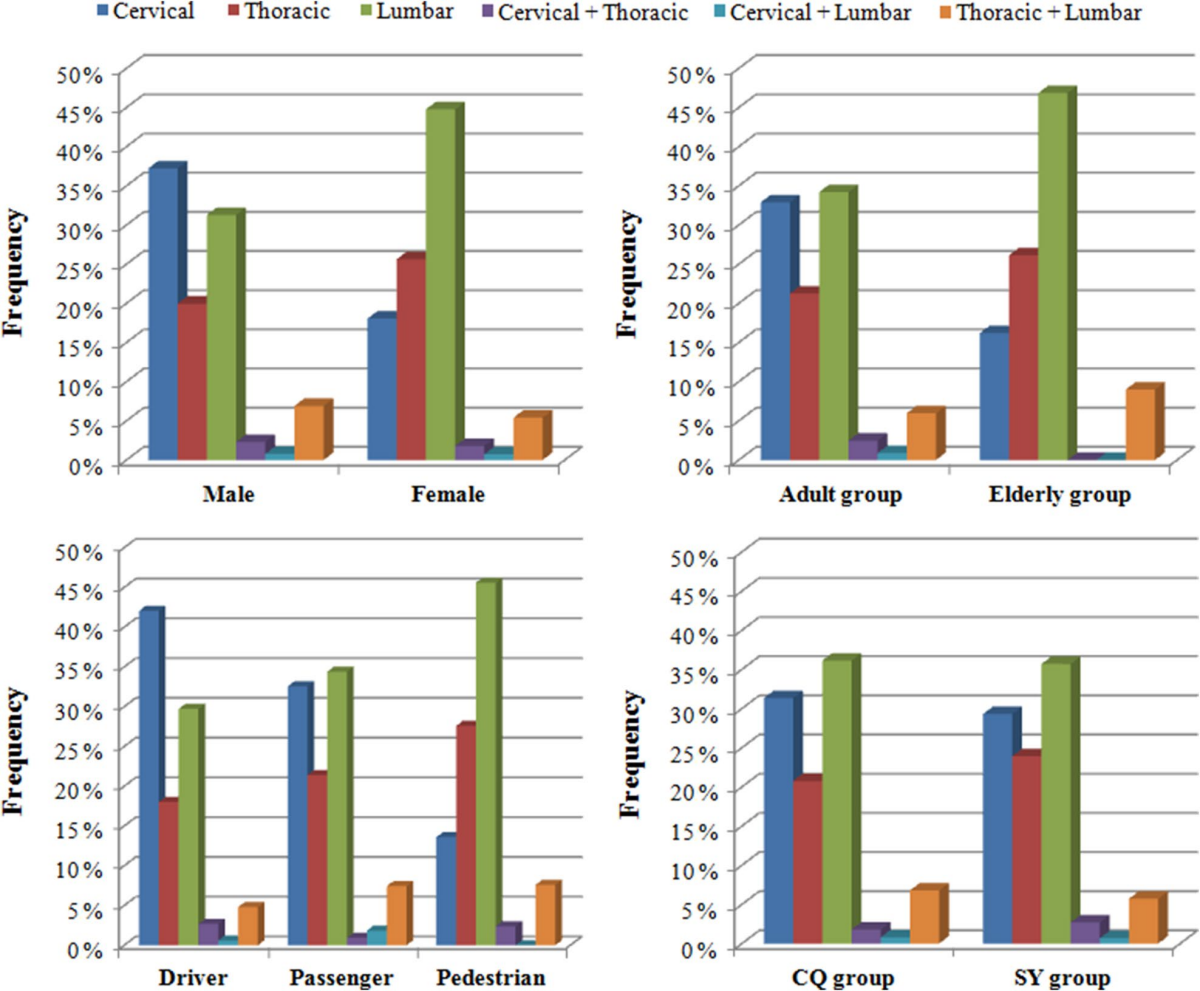
Distributions of fracture locations according to different groups.

**Figure 4:**
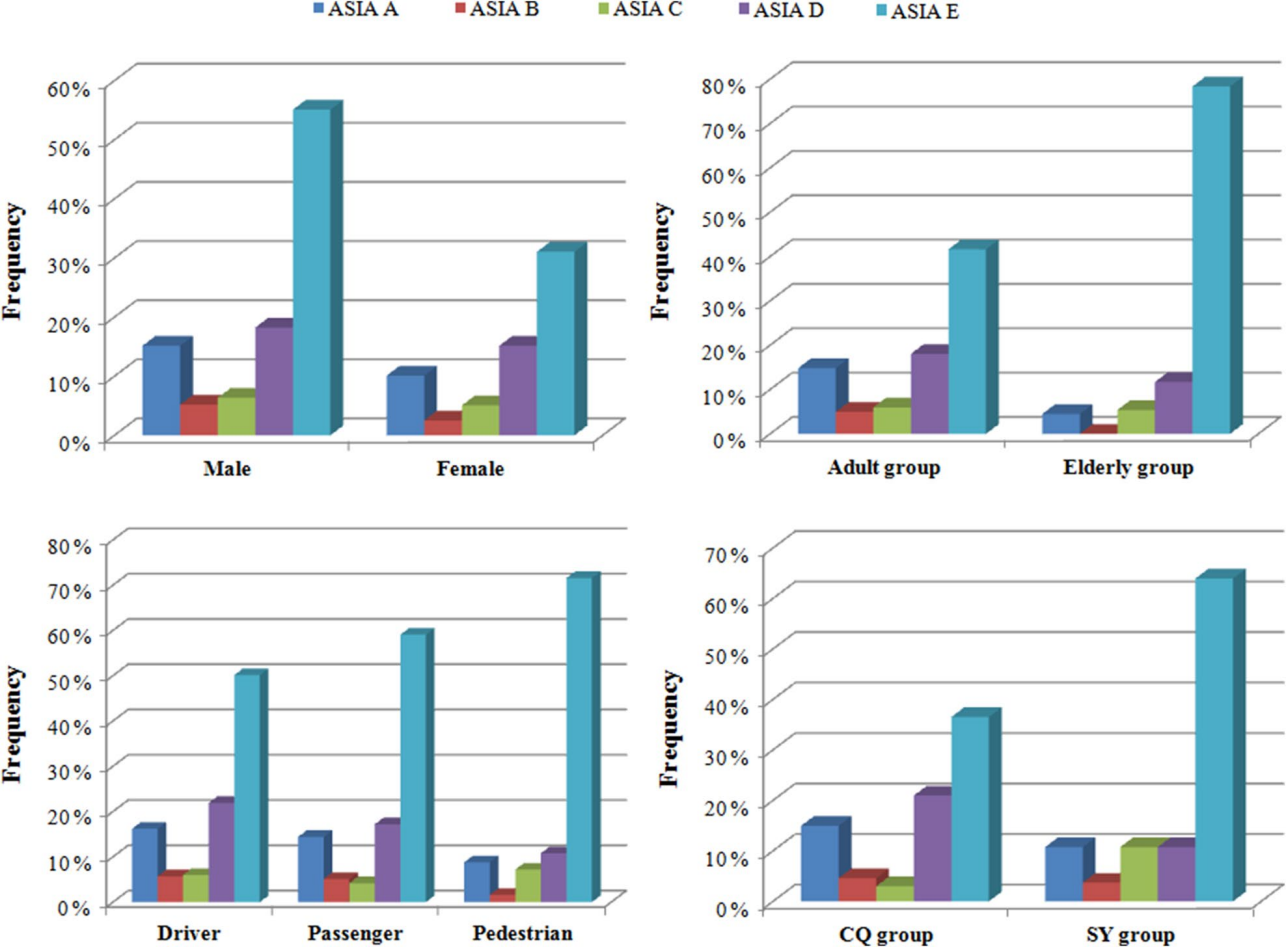
Distributions of spinal cord injury (ASIA classification) according to different groups.

Significant differences in the distribution of district (*p* = 0.018), role (*p* < 0.01), motorcycle (*p* = 0.011), battery electric vehicle (*p* = 0.045), bicycle (*p* = 0.027), coma after injury (*p* = 0.002), pelvic fracture (*p* = 0.021), craniocerebral injury (*p* = 0.008) and fracture location (*p* < 0.01) were observed between the male and female groups. Driver and cervical fracture were observed at a higher frequency among male patients than among female patients. Passenger, pedestrian, thoracic fracture and lumbar fracture were observed at a higher frequency among female patients than among male patients (Table 1) (Figs. 1, 2, 3, 4).

### Age differences in TSFs

Significant differences in the distribution of district (*p* < 0.01), role (*p* < 0.01), car (*p* = 0.013), coma after injury (*p* = 0.003), lower limb fracture (*p* = 0.016), fracture location (*p* = 0.001) and spinal cord injury (*p* < 0.01) were observed between the young adult and elderly groups. Driver, passenger, and cervical fracture were observed at a higher frequency among adult patients than among elderly patients. Pedestrian and lumbar fracture were observed at a higher frequency in elderly patients than in adult patients (Table 2) (Figs. 1, 2, 3, 4).

**Table 2:** Age differences in TSFs caused by MVCs.

Data	Adult group	Elderly group	*p*
Total	687	111	
Mean age (years)	40.7 ± 10.7	67.2 ± 6.1	
Male/Female (sex ratio)	456/231 (2.0)	65/46 (1.4)	0.134
District			
Chongqing	465 (67.7%)	36 (32.4%)	< 0.001
Shenyang	222 (32.3%)	75 (67.6%)	
Injury season			
Spring	163 (23.7%)	31 (27.9%)	0.571
Summer	168 (24.5%)	25 (22.5%)	
Autumn	176 (25.6%)	23 (20.7%)	
Winter	180 (26.2%)	32 (28.8%)	
Role			
Drivers	314 (45.7%)	19 (17.1%)	< 0.001
Passengers	197 (28.7%)	18 (16.2%)	
Pedestrian	176 (25.6%)	74 (66.7%)	
Vehicle involved	797	130	
Car	567 (71.1%)	80 (61.5%)	0.035
Motorcycle	96 (12.0%)	11 (8.5%)	0.299
Truck	36 (4.5%)	8 (6.2%)	0.554
Battery electric vehicle	17 (2.1%)	6 (4.6%)	0.167
Bicycle	23 (2.9%)	8 (6.2%)	0.097
Associated injuries			
LLFs	49 (7.1%)	16 (14.4%)	0.016
PFs	34 (4.9%)	8 (7.2%)	0.448
ULFs	40 (5.8%)	4 (3.6%)	0.468
Craniocerebral injury	86 (12.5%)	16 (14.4%)	0.688
Intrathoracic injuries	183 (26.6%)	31 (27.9%)	0.866
Intra-abdominal injuries	19 (2.8%)	3 (2.7%)	1.000
Coma after injury	45 (6.6%)	17 (15.3%)	0.003
Spinal cord injury	401 (58.4%)	24 (21.6%)	< 0.001
NSFs	85 (12.4%)	16 (14.4%)	0.655
Complications			
Deep venous thrombosis	5 (0.7%)	2 (1.8%)	0.564
Pneumonia	16 (2.3%)	6 (5.4%)	0.127
Pressure sores	4 (0.6%)	0	0.935
Urinary infection	2 (0.3%)	0	1.000
Surgical site infection	3 (0.4%)	0	1.000

### Role differences of TSFs

Significant differences in the distributions of sex ratio (*p* < 0.01), age (*p* < 0.01), district (*p* < 0.01), most vehicles involved (*P* < 0.01), lower limb fractures (*p* < 0.01), pelvic fracture (*p* < 0.01), fracture locations (*p* < 0.01), complications (*p* < 0.01), and spinal cord injury (*p* < 0.01) were observed among the three different groups. Drivers presented with a higher sex ratio. Pedestrians presented with a higher frequency of elderly patients, LLFs and PFs than those in the driver and passenger groups. Pedestrians presented with higher frequencies of thoracic and lumbar fractures and a lower frequency of cervical fracture than drivers and passengers. Drivers and passengers presented with a higher frequency of SCI and a lower frequency of complications than pedestrians (Table 3) (Figs. 1, 2, 3, 4).

**Table 3:** Role differences in TSFs caused by MVCs.

Data	Driver	Passenger	Pedestrian	*P*
Total	333	215	250	
Mean age (years)	40.7 ± 11.6	41.6 ± 12.3	51.6 ± 14.6	
Male/Female (sex ratio)	262/71 (3.7)	125/90 (1.4)	134/116 (1.2)	< 0.001
Age group				
18–60	314 (94.3%)	197 (91.6%)	176 (70.4%)	< 0.001
≥ 60	19 (5.7%)	18 (8.4%)	74 (29.6%)	
District				
Chongqing	225 (67.6%)	154 (71.6%)	122 (48.8%)	< 0.001
Shenyang	108 (32.4%)	61 (28.4%)	128 (51.2%)	
Injury season				
Spring	82 (24.6%)	47 (21.9%)	65 (24.3%)	0.903
Summer	77 (23.1%)	56 (26.0%)	60 (24.2%)	
Autumn	82 (24.6%)	58 (27.0%)	59 (24.9%)	
Winter	92 (27.6%)	54 (25.1%)	66 (26.6%)	
Vehicle involved	424	252	251	
Car	254 (59.9%)	188 (74.6%)	205 (81.7%)	< 0.001
Motorcycle	85 (20.0%)	17 (6.7%)	5 (2.0%)	< 0.001
Truck	19 (4.5%)	14 (5.6%)	11 (4.4%)	0.777
Battery electric vehicle	20 (4.7%)	2 (0.8%)	1 (0.4%)	< 0.001
Bicycle	31 (7.3%)	0	0	< 0.001
Associated injuries				
LLFs	22 (6.6%)	9 (4.2%)	34 (13.6%)	< 0.001
PFs	10 (3.0%)	7 (3.3%)	25 (10.0%)	< 0.001
ULFs	17 (5.1%)	12 (5.6%)	15 (6.0%)	0.895
Craniocerebral injury	40 (12.0%)	23 (10.7%)	39 (15.6%)	0.247
Intrathoracic injuries	81 (24.3%)	60 (27.9%)	73 (29.2%)	0.385
Intra-abdominal injuries	8 (2.4%)	7 (3.3%)	7 (2.8%)	0.836
Coma after injury	20 (6.0%)	16 (7.4%)	26 (10.4%)	0.143
Spinal cord injury	166 (49.8%)	88 (40.9%)	71 (28.4%)	< 0.001
NSFs	32 (9.6%)	33 (15.3%)	36 (14.4%)	< 0.001
Complications				
Deep venous thrombosis	4 (1.2%)	3 (1.4%)	0	0.194
Pneumonia	8 (2.4%)	4 (1.9%)	10 (4.0%)	0.326
Pressure sores	2 (0.6%)	1 (0.5%)	1 (0.4%)	0.940
Urinary infection	0	1 (0.5%)	1 (0.4%)	0.483
Surgical site infection	1 (0.3%)	1 (0.5%)	1 (0.4%)	0.951

### Geographic differences in TSFs

Significant differences in the distributions of sex ratio (*p* = 0.018), age (*p* < 0.01), role (*p* < 0.01), most vehicles involved (*p* < 0.01), coma after injury (*p* = 0.030), LLFs (*P* = 0.002), pelvic fracture (*p* < 0.01), craniocerebral injury (*p* = 0.011), intrathoracic injuries (*p* < 0.01), intra-abdominal injuries (*p* < 0.01), complications (*p* = 0.033) and spinal cord injury (*p* < 0.01) were observed between the Chongqing and Shenyang groups. The patients in the CQ group presented with a higher sex ratio and a higher frequency of adult patient, driver, passenger, SCI and complications than those in the SY group (Table 4) (Figs. 1, 2, 3, 4).

**Table 4:** Geographic differences in TSFs caused by MVCs.

Data	CQ group	SY group	*P*
Total	501	297	
Mean age (years)	41.0 ± 12.1	50.0 ± 14.4	
Male/Female (sex ratio)	343/158 (2.2)	178/119 (1.5)	0.018
Age group			
18–60	465 (92.8%)	222 (74.7%)	< 0.001
≥ 60	36 (7.2%)	75 (25.3%)	
Injury season			
Spring	124 (24.8%)	70 (23.6%)	0.181
Summer	110 (22.0%)	83 (27.9%)	
Autumn	124 (24.8%)	75 (25.3%)	
Winter	143 (28.5%)	69 (23.2%)	
Role			
Drivers	225 (44.9%)	108 (36.4%)	< 0.001
Passengers	154 (30.7%)	61 (20.5%)	
Pedestrian	122 (24.4%)	128 (43.1%)	
Vehicle involved	561	366	
Car	400 (71.3%)	247 (67.5%)	0.216
Motorcycle	91 (16.2%)	16 (4.4%)	< 0.001
Truck	30 (5.3%)	14 (3.8%)	0.364
Battery electric vehicle	0	23 (6.3%)	< 0.001
Bicycle	9 (1.6%)	22 (6.0%)	0.001
Associated injuries			
LLFs	29 (5.8%)	36 (12.1%)	0.002
PFs	14 (2.8%)	28 (9.4%)	< 0.001
ULFs	28 (5.6%)	16 (5.4%)	1.000
Craniocerebral injury	52 (10.4%)	50 (16.8%)	0.011
Intrathoracic injuries	102 (20.4%)	112 (37.7%)	< 0.001
Intra-abdominal injuries	4 (0.8%)	18 (6.1%)	< 0.001
Coma after injury	31 (6.2%)	31 (10.4%)	0.030
Spinal cord injury	318 (63.5%)	107 (36.0%)	< 0.001
NSFs	59 (11.8%)	42 (14.1%)	0.389
Complications			
Deep venous thrombosis	2 (0.4%)	5 (1.7%)	0.137
Pneumonia	11 (2.2%)	11 (3.7%)	0.301
Pressure sores	4 (0.8%)	0	0.305
Urinary infection	0	2 (0.7%)	0.268
Surgical site infection	1 (0.2%)	2 (0.7%)	0.646

## Discussion

Among MVC-related injuries, abdominal and thoracic injuries are serious, resulting in approximately 10% and 25% of MVC-related deaths, respectively^22,23^. Thoracic and abdominal injuries increase the risk of spinal injuries^24^. Thoracic injuries, especially dorsal spinal injuries, commonly accompany TSFs. Thoracic injuries account for 41% of TSF patients and are considered to be the most common TSF-associated injuries^7^. The in-depth analysis of the association discussed above is more important following MVCs because MVCs result in more TSF-associated injuries than situations involving lower energy mechanisms^7,25,26^. This in-depth analysis will contribute to a better understanding of the disease and more effective prevention of injury.

Gender plays an important role in the pattern of TSFs resulting from MVCs. Male patients were more frequently associated with driver, cervical fracture, and CCI than female patients. Female patients were more frequently associated with passenger, pedestrian, thoracic and lumbar fracture, and SCI than male patients. A previous study showed that thoracic and abdominal injuries accounted for 10.5% of MVC-related TSFs, and male patients with MVC-related TSI were more frequently associated with thoracic- and abdominal-associated injuries than females^16^. In the current study, the number of male patients exceeded the number of female patients, a phenomenon that could be explained by gender demographics and the Chinese culture; men are involved in more social activities than women, and women rarely drive motorcycles.

Age also plays an important role in the pattern of TSFs resulting from MVCs. Adult patients were more frequently associated with driver, passenger, cervical fracture and SCI than elderly patients. Elderly patients were more frequently associated with pedestrian and lumbar fracture than adult patients. Thoracic and lumbar spine fracture patterns are influenced by the age of the occupant and the type and use of seat belts. Extension injuries occur in older obese individuals and are associated with a high fatality rate^20^. Previous studies have shown that established risk factors for fatal teen driver crashes, including restraint nonuse, transporting teen passengers, and speeding, also increase the risk of nonfatal injury in single vehicle crashes^27^. Thus, more attention should be given to the timely diagnosis and treatment of cervical fracture and spinal cord injury among young patients, especially when the patient is rushed to the hospital for emergency treatment.

MVC patients with different roles, such as driver, passenger or pedestrian, exhibit different distributions of injury characteristics, and the phenomenon is directly related to the population studied and the local traffic situation^28–31^. Head injuries account for most pedestrian fatalities in crashes with powered 2-wheelers in India, and lower extremity injuries account for most nonfatal injuries^28^. Older pedestrians, male drivers, older drivers and intoxicated motorists are prevalent determinants of pedestrian fatalities in glare-related crashes^29^. Enhancing the conspicuity of pedestrians with the use of visibility aids may be beneficial for reducing crash risk or severity^30^. Pedestrians may be at increased risk of injury when the driver is male, when the driver is under the influence of alcohol, when the pedestrian is struck while in the travel lane, when the pedestrian is aged 65 or older, and when the pedestrian is under the influence of alcohol^31^.

In the current study, drivers, who presented with the highest sex ratio (3.7), accounted for 41.7% of TSFs caused by MVCs. Pedestrians were observed at a higher frequency among elderly patients, LLFs and PFs than drivers and passengers. Pedestrians exhibited a higher frequency of thoracic and lumbar fractures and a lower frequency of cervical fractures than drivers and passengers. Drivers and passengers exhibited SCIs at a higher frequency than pedestrians. Passengers presented with fewer lower extremity fractures due to the protective nature of the car cage. It has been shown that pedestrians are prone to CCIs, ITIs, LLFs and PFs due to multiple impacts. We should provide advanced life support measures at the scene, stabilize the airway and protect the entire spine of pedestrians. NSFs were observed at a greater frequency among passengers and pedestrians than among drivers. Much more attention should be given to passengers and pedestrians, especially if NSFs are found, to avoid a missed and delayed diagnosis. Enhancing vehicle safety features for pedestrians, forbidding driving and walking while intoxicated, investigating local pedestrian injury trends and making progress with public health prevention strategies will play important roles in reducing the risk and severity of crashes^32,33^. Advance-warning signs of TSFs caused by MVCs may provide information to policy-makers for educational efforts and facilitate the establishment of suitable and effective policies and strategies.

The use of motor vehicles is rapidly increasing in Chongqing, which is a mountainous city. Because of a lack of strict traffic safety regulations, the probability of sustaining a serious trauma caused by MVCs is high. Shenyang is located in the cool northern region of China, extending mainly to the plains, and the frequency at which pedestrian was observed was significantly higher in the SY group. Significant differences were observed in the distributions of age, role, motorcycle, battery electric vehicle, bicycle, taxi, coma after injury, LLFs, PFs, CCI, ITIs, IAIs, complications and SCIs between the CQ group and SY group. A previous study showed that India is a highly populous country with a significant number of powered 2-wheelers in its traffic mix. This mix and the density of both pedestrians and PTWs translate to a high potential for unsafe interactions between the 2 types of road users^28^. The empirical results that sun glare is a combined spatiotemporal factor associated with pedestrian fatalities may be unique to Taiwan because of its unique sunrise and sunset times and orientations^29^. Younger patients, especially those with MVCs involving motorcycles in Chongqing, should be given more attention to prevent nerve injuries. In Shenyang, more attention should be given to patients who present with associated injuries for the timely diagnosis and treatment of serious associated injuries.

### Limitations

This study had some limitations. First, the retrospective design and the small number of patients may have resulted in selection bias. Second, there was a lack of information about bone mineral density. Despite these limitations, we believe that this valuable epidemiological information can be used as guidance for the prevention of TSFs caused by MVCs.

## Conclusions

This study demonstrates the age-, gender-, role- and geographic-specific clinical characteristics of TSFs resulting from MVCs and reveals a significant relationship between different ages, sexes, roles, geographic locations and associated injuries, complications and spinal cord injury.

## Data Availability

The data that support the findings of this study are available from the corresponding author upon special request.
